# Costas Stefanis MD: Formerly Professor of Psychiatry and Head, Department of Psychiatry, University of Athens Medical School; Director, Eginition University Psychiatric Hospital, Athens

**DOI:** 10.1192/pb.bp.116.055871

**Published:** 2017-10

**Authors:** Nick Bouras

**Figure:**
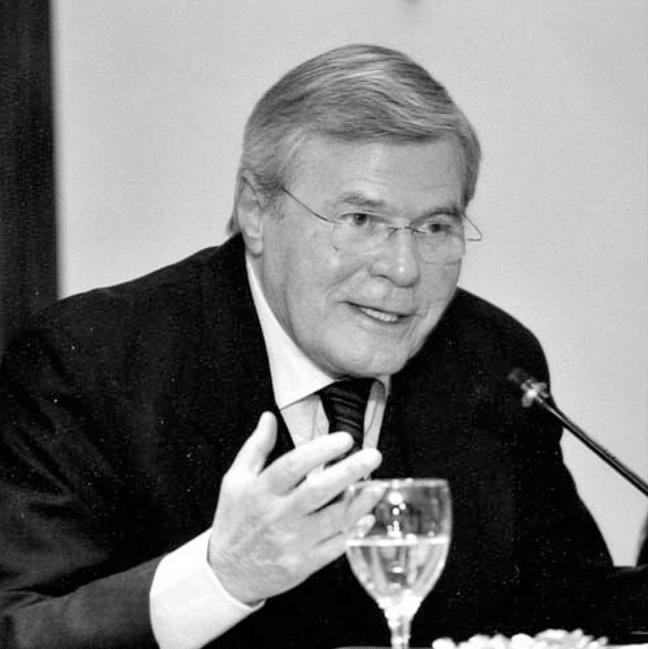


Costas Stefanis, who died recently at the age of 88, was the leading figure in psychiatry in Greece for 30 years, from the time of his appointment as professor of psychiatry in 1970. In collaboration with British-trained colleagues he transformed Greek psychiatry and mental health services, steering away from a previously narrow traditional psychoanalytic approach towards a much broader biopsychosocial direction. He expanded the provision of generic and specialist mental health services and was quick to adopt the new emerging trends of community care by developing the first community mental health centre in Greece. He developed a strong research programme, having himself – early in his career, while in the USA and Canada – carried out pioneering work on the functional role of pyramidal neurons in the sensorimotor cortex. He had an international reputation as an expert on the mode of action of neurotransmitters functioning on central nervous system synapses.

Several generations of Greek psychiatrists and allied professionals were trained by him, including me. Many became his associates and were inspired to go on to acquire international recognition and reputation. In 1989, in Athens, he founded the University Mental Health Research Institute – which undertook much neurobiological and psychosocial research into mental disorders – and remained president and director until his death.

He also played a major part in international psychiatry. As president of the World Psychiatric Association (WPA) from 1983 to 1990, he was responsible for an organisation in crisis. The political use of psychiatry in the Soviet Union to incarcerate political dissidents on the grounds of mental illness led to an explosive climate within the WPA. In 1983, after major criticism from societies in other countries – which made it likely that it was facing expulsion – the All-Union Society of Psychiatrists and Neuropathologists of the USSR withdrew from the WPA. Years later, at the WPA congress held in Athens in 1989, the general secretary of that Society publicly acknowledged that political abuse of psychiatry had indeed taken place and the organisation was reinstated as a member. This by no means ended the unhappy situation in the Soviet Union, which to some extent continues to this day, but a greater degree of openness was achieved. Costas Stefanis was among those responsible for reaching a conciliatory approach on issues with significant ethical and political dimensions.

Costas Stefanis became active in the political life of his country. Between 1996 and 2000 he served as honorary member of the Greek Parliament in the reformist social-democratic government and was Minister of Health and Welfare from 2002 to 2004. During his ministerial tenure, he was president of the Council of Ministers of Health of the European Union and succeeded in achieving approval for anti-stigma legislation regarding mental illness. On behalf of the EU member states, he signed the World Health Organization's International Treaty on Tobacco Advertising. In Greece, he was responsible for four major bills passed by Parliament – on public health; reform and decentralisation of health services; the organisation of primary health care and provisions for prevention and social reintegration. Unfortunately, most of them are still awaiting implementation.

He received numerous distinctions and awards. In 1994 he was elected life member of the Athens Academy of Sciences and Arts – the highest level of scientific recognition in Greece – and served as its president in 2006. He was awarded the Medal of the Cross of the President of the Hellenic Republic in recognition of his distinguished service to the country. In acknowledgement of his contribution to psychiatry, the World Federation of Societies of Biological Psychiatry and International Neuropsychiatric Association established an international prize: the Costas Stefanis Award for Excellence in Psychiatry and the Neurosciences. He was elected honorary member and fellow of several scientific associations and he authored numerous peer-reviewed articles as well as books and articles in the lay press.

Costas Stefanis was born in Greece in 1928 and graduated from the Medical School of Athens University in 1953. He trained in neurology and psychiatry in Athens and subsequently served as a fellow in basic neurophysiology at McGill University, Montreal, and as a research scientist at the National Institute of Mental Health and Saint Elizabeth Mental Hospital (Bethesda, Maryland and Washington, DC).

He was a highly intelligent, conscientious professional who, during difficult times, strove to improve psychiatric and mental health service provision not only in Greece but worldwide, thus improving the lives of thousands of people. He died on 29 October 2016 after a long illness stoically borne, remaining active in offering advice and ideas to the end. He was devoted to his family and is survived by his wife Adela, two sons – Nicos, professor of psychiatry, and Leonidas, professor of neurology – his daughter Evanthia, a film maker, and four grandchildren.

The death of Costas Stefanis will be felt as a great loss to psychiatry by many clinical, academic and international colleagues who had the privilege of knowing and working with him.

